# Mixed Primary Cultures of Murine Small Intestine Intended for the Study of Gut Hormone Secretion and Live Cell Imaging of Enteroendocrine Cells

**DOI:** 10.3791/55687

**Published:** 2017-04-20

**Authors:** Arianna Psichas, Gwen Tolhurst, Cheryl A. Brighton, Fiona M. Gribble, Frank Reimann

**Affiliations:** ^1^Metabolic Research Laboratories and MRC Metabolic Diseases Unit, Wellcome Trust-MRC Institute of Metabolic Science, University of Cambridge

**Keywords:** Cellular Biology, Issue 122, Small intestine, duodenum, mouse, primary, culture, enteroendocrine, L cell, gut, hormone

## Abstract

The gut is the largest endocrine organ of the body, with hormone-secreting enteroendocrine cells located along the length of the gastrointestinal epithelium. Despite their physiological importance, enteroendocrine cells represent only a small fraction of the epithelial cell population and in the past, their characterization has presented a considerable challenge resulting in a reliance on cell line models. Here, we provide a detailed protocol for the isolation and culture of mixed murine small intestinal cells. These primary cultures have been used to identify the signaling pathways underlying the stimulation and inhibition of gut peptide secretion in response to a number of nutrients and neuropeptides as well as pharmacological agents. Furthermore, in combination with the use of transgenic fluorescent reporter mice, we have demonstrated that these primary cultures become a powerful tool for the examination of fluorescently-tagged enteroendocrine cells at the intracellular level, using methods such as patch clamping and single-cell calcium and cAMP-FRET imaging.

**Figure:**
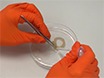


## Introduction

The overall goal of this method is to isolate and culture mixed murine intestinal cells to enable the study of gut peptide secretion and live cell imaging of enteroendocrine cells. An early version of this procedure was originally published in 2008 by Reimann *et al*.^1^ and has since formed the basis of a further 20 publications from our group. For this manuscript, we chose to focus on small intestinal cultures as they are more challenging to establish than colonic cultures.

Enteroendocrine cells secrete an array of gut peptides including glucagon-like peptide 1 (GLP-1), glucose-dependent insulinotropic peptide (GIP), cholecystokinin (CCK) and peptide YY (PYY)^2^. These gut peptides have important physiological roles in orchestrating postprandial responses such as the slowing of gut transit, potentiation of glucose-stimulated insulin release and induction of satiety. GLP-1 mimetics and drugs which inhibit its degradation are currently licensed for the treatment of type 2 diabetes and there is ongoing assessment of the therapeutic potential of stimulating the endogenous secretion of this peptide^3^. A better understanding of enteroendocrine physiology and the mechanisms by which secretion is either stimulated or inhibited is, therefore, of critical importance.

Gut peptide secretion can be studied using a variety of *in vitro* and *ex vivo* experimental models, each with advantages and disadvantages (for a comprehensive recent review also covering the use of *in vivo* models see Svendsen *et al*.^4^). *Ex vivo* techniques such as the isolated perfused intestine^5^ and Ussing chambers^6^ are currently being used to explore gut peptide secretion in response to various substances. The main advantage of these methods, as compared to primary cultures, is that the immediate environment of the enteroendocrine cells remains largely intact and importantly, the polarity of the cells is preserved. Therefore, it is possible to elicit information regarding which cell surface a secretagogue is acting on^6^. However, these are typically low-throughput techniques and the quality of the data derived from them relies heavily on the integrity and viability of the intestinal tissue, which inevitably decline over time.

Intestinal organoids are now increasingly being used as *in vitro* models for the study of enteroendocrine cell function and development^7^. Organoids, which can be derived from both murine and human tissue, have the benefit of forming 3D 'crypt-like' structures that maintain cell polarity in culture. However, organoid-derived enteroendocrine cells have yet to be fully characterized and their similarity to native L cells remains largely unknown. Primary cultures have the advantage of being a step closer to the physiological setting, as enteroendocrine cells are not maintained and differentiated in artificial conditions for prolonged periods of time.

An alternative primary culture method which has been employed for the assessment of gut peptide secretion is the isolation of fetal rat intestinal cells (FRICs)^8^. However, the culture of adult intestinal tissue has met with less success and some differences have been observed in the responsiveness of FRICs *vs.* adult murine intestinal cells (*e.g.* glucose^8^).

Enteroendocrine-like cell lines (*e.g.* GLUTag, STC-1 and NCI-H716) have traditionally been used to distinguish direct *vs*. indirect effects on enteroendocrine cells and to dissect underlying molecular mechanisms coupling stimulus to secretion; see Kuhre *et al*.^9^ for peptide production and secretion by 'L cell-like' immortalized cell lines. This has been necessary as enteroendocrine cells, scattered along the gastrointestinal tract, constitute just under 1% of intestinal epithelial cells^10^ and sorted cells, in our hands, do not survive in culture^1^. Cell lines remain useful tools owing to the fact that they are a largely homogeneous cell population which is conducive to genetic manipulations such as gene-silencing. Consequently, it is easy to investigate the role of proteins which cannot be targeted pharmacologically, when transgenic mice are not available. However, cell lines are not always valid models of primary cells. While there are many similarities, findings from primary cultures have on occasion highlighted differences in the signaling pathways activated by certain nutrients in primary L cells compared to GLUTag cells, for example (*e.g*. peptones^11^). Crucially, in combination with transgenic fluorescent reporter mice, the primary culture model enables detailed examination of individual primary enteroendocrine cells at the intracellular level. Fluorescently-tagged L cells within primary cultures have been used by our group for patch clamping^1^,^12^ studies, and single-cell calcium^11^,^13^ and cyclic adenosine monophosphate-FRET imaging^14^ studies, which have yielded significant advances in the field of enteroendocrine physiology.

The following protocol is optimized for performing secretion experiments, using a 24-well plate or for the preparation of up to 16 imaging dishes, from a 10 cm section of the small intestine from a single adult mouse. The protocol can be easily modified for the study of colonic cells by increasing digestion times and collagenase concentration.

## Protocol

All animal procedures were approved by the University of Cambridge Animal Welfare and Ethical Review Body and conformed to the Animals (Scientific Procedures) Act 1986 Amendment Regulations (SI 2012/3039).

### 1. Preparation in Advance

1. Place an aliquot of basement membrane matrix (BMM) (~200 µL) on ice to thaw. NOTE: The BMM solidifies at room temperature (RT).
2. Pre-warm 50 mL sterile high-glucose Dulbecco's Modified Eagle Medium (DMEM) (with no additions) and ~40 mL sterile culture medium (high-glucose DMEM supplemented with 10% Fetal Bovine Serum (FBS), 100 U/mL penicillin and 0.1 mg/mL streptomycin (P/S), and 2 mM L-glutamine) in 50 mL centrifuge tubes in a water bath at 37 ^o^C.
3. Pre-chill calcium and magnesium-containing Phosphate-Buffered Saline (PBS).
4. **Weigh out 15 mg collagenase (XI crude), add to a 50 mL centrifuge tube and keep it on ice.**
  1. CAUTION: Collagenase is harmful. It causes skin irritation (H315) and serious eye irritation (H319). It may cause allergy or asthma symptoms or breathing difficulties if inhaled (H334). It may cause respiratory irritation (H335). Use appropriate personal protective equipment, avoid dust formation and avoid breathing dust. Ensure adequate ventilation.

### 2. Tissue Collection

1. Add ~20 mL L-15 medium to a 50 mL centrifuge tube and place it on ice.
2. Euthanize a mouse by cervical dislocation, or other approved schedule 1 method. NOTE: Intestinal tissue is typically obtained from mice on a C57BL6 background. However, other genetic backgrounds have also been used *e.g.* 129/SvEv^15^. Imaging experiments require the use of fluorescent reporter mice *e.g.* GLU-Venus^1^. Tissue is obtained from adult mice (2-6 months) of both sexes. The mice are housed in individually-ventilated cages with *ad libitum* access to water and regular chow. However, experiments to test the effect of a high-fat diet, for example, on enteroendocrine cell function^16^ can be performed if supported by a project license.
3. Dissect and gently remove mouse intestine (from pylorus to start of rectum) using forceps and dissection scissors. Store it in L-15 medium on ice until ready to use.

### 3. Tissue Preparation

1. Place intestinal tissue in a 10 cm petri dish containing enough PBS to cover the tissue. Take 10 cm of desired tissue (*e.g.* upper small intestine, top 10 cm, distal to pylorus).
2. **Flush out intestinal contents using a plastic Pasteur pipette and chilled PBS.**
  1. Using forceps, delicately grip one end of the intestinal segment and place the tip of a Pasteur pipette into it. Flush out contents with chilled PBS. Repeat from both ends until the majority of the contents has been flushed out. Transfer to a clean petri dish containing fresh chilled PBS.
3. Using forceps, remove the adipose tissue and mesentery, taking care to not pull off the muscle layer at the same time.
4. **Peel the muscle layer off "like a sock" under a dissecting microscope using two sets of fine forceps.** NOTE: This step is not essential but is highly recommended. There are alternative ways of removing the muscle layer to the one described here. For example, the intestine can be cut open longitudinally first, prior to the removal of the muscle layer.
  1. Find a starting point at the more proximal end of the tissue where there is a visible flap of muscle. Gently pull away a small amount of muscle layer all the way around the intestine. NOTE: To avoid tearing of the muscle layer or the intestinal epithelium, reduce the tension force by clamping a larger surface area rather than using the tips of the fine forceps.
  2. Clamp the intestine and as much of the muscle flap as possible, gently pull apart and start peeling off the muscle layer from around the intestine. To avoid tearing both the muscle layer and epithelium, keep readjusting the position of the forceps to keep them close together. In this way, remove the muscle layer from the entire length of the intestinal segment and discard.
5. Cut the intestine open longitudinally and wash by swirling in a clean petri dish with fresh chilled PBS. Repeat if necessary to remove any remaining chyme or mucus.
6. Mince tissue with a surgical scalpel blade to achieve squares of ~1-2 mm^2^ and add these to ~20 mL chilled PBS in a 50-mL centrifuge tube using a Pasteur pipette. To avoid tissue pieces sticking to the pipette, cut off the tip and wet the pipette by triturating with PBS.
7. Gently shake the tube to further wash the tissue pieces. Allow the tissue to settle and pour or pipette off the majority of the PBS and repeat with fresh PBS until the PBS looks clear.

### 4. Preparation of BBM-coated Plate/Dishes and Digestion Medium NOTE: The following steps should be performed in a tissue culture hood (with incubation steps in 37 °C water bath).

1. **Prepare a 2% BMM solution in chilled DMEM (with no additions). While working, keep the solution on ice. For a 2% solution, add 140 µL thawed BMM to 7 mL DMEM (enough to prepare a single 24-well plate).**
  1. Add 250 µL 2% BMM solution per well (24-well plate) or per glass bottom imaging dish.
  2. Incubate coated plates/dishes for at least 30 min in an incubator at 37 °C to allow for adequate polymerization of the BMM.
2. **Add the 50 mL pre-warmed DMEM (with no additions) to the 15 mg collagenase (from Steps 1.2 and 1.4) to form a 0.3 mg/mL solution and invert to dissolve.**
  1. Once completely dissolved, using a 20 mL syringe, filter the collagenase solution through a 0.2 µm sterile filter into a new sterile 50 mL centrifuge tube. Label this as the digestion medium.

### 5. Tissue Digestion

1. **Remove the tissue pieces from the PBS using a 10 mL serological pipette and add to a sterile 50-mL centrifuge tube containing chilled sterile DMEM (with no additions), swirl and then remove DMEM.** NOTE: To avoid tissue sticking to the serological pipette, wet it by trituration with DMEM prior to contact with the tissue.
2. **'Digest' 1 and 2: Removal of cell debris and single cells**
  1. Add 7 mL digestion medium to the tissue pieces and give the tube a swirl.
  2. Incubate for 5 min in a water bath at 37 °C.
  3. Gently shake (not swirl) the tube for ~3 s.
  4. Allow the tissue to settle and discard the digestion medium, reserving a small volume to look at under the microscope. NOTE: Especially when starting out, it is highly recommended to inspect a small volume of each 'digest' (~30 µL) under the microscope to gauge the progress of the digestion process. If many crypts are observed at this stage, the shaking may be too vigorous. For representative images of what the different 'digest' stages typically look like, refer to **Figure 1**.
  5. Repeat Steps 5.2.-5.2.4 (with fresh digestion medium).
3. **'Digests' 3 to 5: Collection of crypt fragments**
  1. Add 7 mL digestion medium to the tissue.
  2. Incubate for 10 min at 37 °C.
    1. During the incubation, shake every 5 min for 10-12 s. NOTE: The shaking is more vigorous than the shaking for 'digests' 1 and 2.
  3. Allow undigested tissue to settle and collect the digestion medium in a 15 mL centrifuge tube. If tissue is accidentally collected along with the medium, allow it to settle, remove it using a 10 mL serological pipette and transfer it back to the 50-mL centrifuge tube containing the undigested tissue.
  4. Centrifuge collected medium/supernatant at RT for 3 min at 100 x g.
  5. Discard the supernatant and re-suspend the cell pellet in 5 mL pre-warmed culture medium by gentle trituration and set aside.
  6. Inspect a small volume of the cell suspension under the microscope (see Note from Step 5.2.4). NOTE: Ideally, crypt fragments start to appear in 'digest' 3 along with cell debris and single cells. 'Digests' 4 and 5 contain a significantly greater number of crypt fragments with reduced cell debris (**Figure 1**). Adjust shaking intensity as necessary *i.e.* if the tissue is not digesting and crypt fragments are not appearing, shake more vigorously.
  7. Repeat steps 5.3.1-5.3.6 until 5 'digests' have been completed or until the majority of the tissue has been digested (a 6^th^ digest may be required).
  8. Once all supernatants from 'digests' 3-5 (or 3-6) have been collected, centrifuge the digest supernatants at RT for 3 min at 100 x g.
  9. For secretion experiments, combine the supernatants from 'digests' 3-5 (or 3-6) prior to centrifugation. NOTE: Less tissue is needed for imaging experiments, therefore choose the digest supernatant that is the cleanest (absence of cell debris and single cells) with the greater number of crypt fragments. This is typically 'digest' 4 or 5.
  10. Discard the supernatant and gently re-suspend the pellet by triturating until no clumps are visible. Re-suspend the pellet in pre-warmed culture medium supplemented with 10 µM Y-27632 dihydrochloride (to prevent anoikis^17^). Use 5 mL for secretion experiments and 2 mL culture medium for imaging experiments.
  11. Filter cell suspension through a 100-µm filter (to remove any undigested tissue). Run a further 2 mL of pre-warmed culture medium through the filter to wash (total volume: 7 mL and 4 mL for secretion and imaging, respectively). NOTE: Filtering is not essential, however, larger tissue fragments tend not to adhere to the plate/dishes.

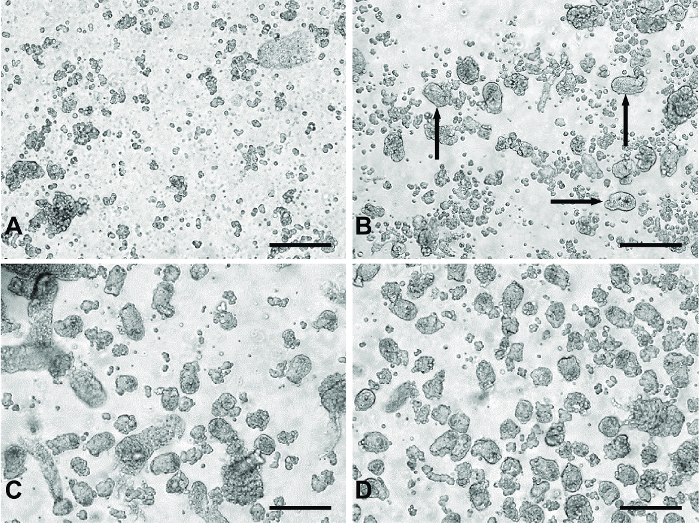
**Figure 1: Representative images of 'digests' from primary small intestinal culture method. **(**A**) Typical material from 'digests' 1 and 2 containing primarily single cells and cell debris. (**B**) An example of products from 'digest' 3. The black arrows indicate crypt fragments appearing in the digest material. (**C**) Typical of 'digest' 4 or 5. Panel (**D**) represents pooled digest material from 'digests' 3-5 passed through a 100 µm filter to remove the larger unwanted tissue fragments seen in (**C**). All images were taken using a digital inverted microscope with a 20X objective. Scale bars = 100 µm. Please click here to view a larger version of this figure.

### 6. Plating Intestinal Cultures (Enriched for Crypt Cells)

1. Remove excess BMM solution from plate or dishes, and add 250 µL pre-warmed culture medium per secretion well. NOTE: Imaging dishes are left without medium so proceed to the next step swiftly to prevent the dish from drying out.
2. **Plate 250 µL cell suspension per well (24-well plate) or per glass bottom dish.**
  1. Plate drop-wise in a slow 'zig-zag' motion across the well. Allow crypt fragments to settle for ~5 min before moving the plate to the incubator. NOTE: This should encourage an even distribution of crypts/cells within the well.
3. Incubate plate or dishes overnight at 37 °C and 5% CO_2_. NOTE: A 'patchy' monolayer should have formed. For a representative image of cultures following three washes, see **Figure 2A**.
4. 'Flood' imaging dishes with 2 mL pre-warmed culture medium per dish. NOTE: These dishes will be suitable for imaging for up to ~72 h following plating. Colonic cultures typically last longer, up to 7 days. Cells which have not adhered can be removed by a wash step. Primary cultures are now ready to be used for experiments.

## Representative Results

The use of serial digestion steps allows the collection of a relatively clean preparation of crypt fragments to be plated for experiments. The first two digests remove primarily single cells and cell debris from the digesting material (**Figure 1A**). During the third digest, crypt fragments appear in the digested material (**Figure 1B**). Digests 4 and 5 yield a greater number of crypt fragments with fewer single cells (**Figure 1C**). Filtering the pooled material from digests 3-5 removes larger pieces of undigested tissue, which may be detrimental to the culture, producing a clean crypt fragment preparation (**Figure 1D**).

Following 18-24 h of culture, monolayers of primary small intestinal cells are observed (**Figure 2A**). Using cultures generated from transgenic mice specifically expressing the calcium fluorescent sensor, GCaMP3, under the control of the proglucagon promoter^11^, L cells are readily identifiable and interspersed within the culture (**Figure 2B**). In primary small intestinal cultures, compounds targeting the G_q_-Ca^2+^_i _and cAMP_i_-dependent pathways stimulated the secretion of GLP-1 (for secretion experiment methodology see Reimann *et al*.^1^). Bombesin (BBS, 100 nM) and co-application of forskolin and 3-isobutyl-1-methylxanthine (IBMX) (F/I, 10 µM each) triggered a 2- and 11-fold, stimulation of GLP-1 release relative to basal, respectively (**Figure 2C**). Specific L cell expression of GCaMP3 allows the identification of and real-time monitoring of calcium mobilization in individual L cells. Both 100 nM bombesin (BBS) and 30 mM potassium chloride (KCl) stimulated transient increases in intracellular calcium indicative of the known G_q_- and electrogenic-coupled pathways in primary L cells (**Figure 2D**).


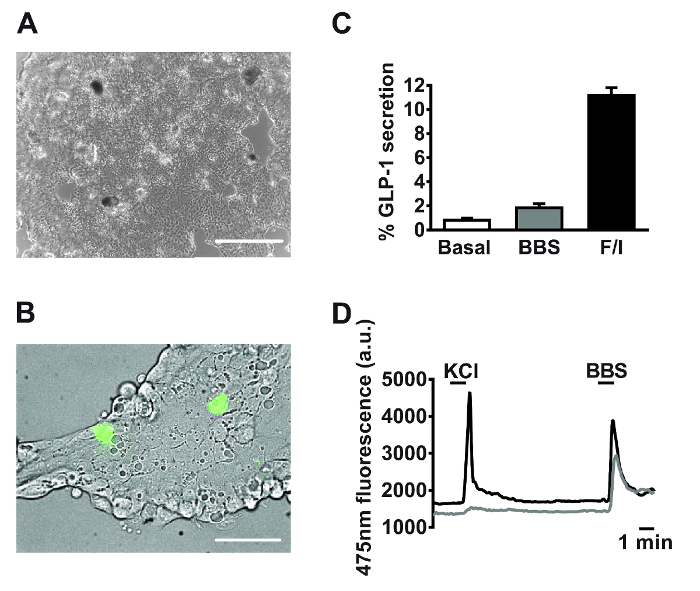
**Figure 2: Representative data derived from primary small intestinal cultures. **Example images of mixed primary small intestinal cultures used for (**A**) secretion and (**B**) imaging experiments post 24 h plating. (**A**) Image taken from a 24-well plate, using a digital inverted microscope with a 4X objective. Scale bar = 500 µm. (**B**) Using GLU-Cre x ROSA26 GCamP3 mice, L cells in the field of view (green cells) were identified by the fluorescence of GCaMP3. Scale bar represents 50 µm. (**C**) GLP-1 secretion was measured in response to bombesin (BBS, 100 nM) and forskolin/3-isobutyl-1-methylxanthine (IBMX) (F/I, 10 µM each). Percentage GLP-1 secretion was calculated by measuring GLP-1 levels in the supernatants and cell lysates. Data represent the means ± SEM of n = 3 for each condition. (**D**) GCaMP3 fluorescence (reflecting cytosolic calcium) of the two cells outlined in (**B**) monitored in real time in response to potassium chloride (KCl, 30 mM) and BBS (100 nM). Single cell imaging was performed using an inverted fluorescence microscope with a 40X oil-immersion objective. GCaMP3 was excited at 475/10 nm, using a 75 W xenon arc lamp and a monochromator controlled by fluorescence imaging software. Emission was recorded with a high-resolution digital charge-coupled device (CCD) camera using a dichroic mirror and a 510-560 nm bandwidth filter. Please click here to view a larger version of this figure.

## Discussion

This protocol describes the isolation and culture of mixed murine small intestinal cells to enable the study of gut peptide secretion and single cell imaging of labelled enteroendocrine cells.

To increase the likelihood of the small intestinal cultures being successful, it is important that the protocol is carried out as swiftly as possible (ideally, within 3 h of harvesting the tissue) and that the tissue is kept in ice-cold medium or buffer prior to the digestion process, to limit cell death. While it is not essential, especially for colonic cultures, removal of the muscle layer from the small intestine is strongly encouraged. The small intestinal culture protocol has been standardized as much as possible. However, it is important to note that no digestion process is identical. There are many steps during this protocol where variability can be introduced on a day-to-day basis *e.g.* the degree of muscle removal, the size of the 'minced' tissue pieces, the strength of shaking, the potency of a particular batch of collagenase *etc*. It is therefore, of paramount importance to inspect aliquots of each of the different 'digests' under the microscope to assess the progress of the digestion process and tailor the shaking and the number of 'digests' accordingly. It is recommended to shake more gently to begin with and, if crypt fragments are not obvious from 'digest' 3 onwards, to start shaking more vigorously.

From our experience, enteroendocrine cells do not proliferate in culture and are usually lost from small intestinal cultures within 4 days. Nevertheless, this allows ample time to perform gut peptide secretion experiments (typically carried out over 2 h, 18-24 h post plating) to test physiological stimuli and/or pharmacological agents. Experiments requiring an overnight incubation prior to the secretion experiment are also feasible *e.g.* in the case of pre-treatment with pertussis toxin^15^.

The primary culture technique presented here is a well-established method, as evidenced by the volume of research output it has produced over the years. The primary culture model has been used to study the secretion of a variety of gut peptides, including GLP-1^1^, GIP^18^ and PYY^19^, in response to diverse nutrient and non-nutrient^14^,^20^ stimuli and inhibitors. Furthermore, the same technique has been successfully applied to the culture of human intestinal cells^21^. Often, a combination of the primary cell culture method along with an additional experimental model *e.g*. *ex vivo* or *in vivo* can provide the most insight^6^. Notably and in contrast to other methods, this technique has important applications beyond the measurement of gut peptide release. We have demonstrated that primary small intestinal cultures derived from transgenic reporter mice are powerful tools for the interrogation of intracellular signaling pathways coupled to gut peptide secretion, using for example the Ca^2+^ and cAMP sensors, GCaMP3^11^,^13^ and Epac2camps^14^, respectively, as well as electrophysiological techniques^12^.

As with all *in vitro* models, the primary intestinal cultures have certain inherent limitations including the loss of polarity of the epithelial cells in culture, as well as the loss of blood supply and innervation. It is difficult to estimate the potential impacts of these artificial conditions on enteroendocrine cell function. However, data obtained from primary cultures have often been translatable in the *in vivo* setting (*e.g.* effects of short-chain fatty acids on GLP-1 secretion^15^).

The primary intestinal cultures are a versatile technique with many potential applications. They have already provided important mechanistic insight into the regulation of gut peptide secretion.

## Disclosures

The authors have nothing to disclose.
